# VX-166: a novel potent small molecule caspase inhibitor as a potential therapy for sepsis

**DOI:** 10.1186/cc8041

**Published:** 2009-09-09

**Authors:** Peter Weber, Ping Wang, Stephane Maddens, Paul SH Wang, Rongqian Wu, Michael Miksa, Weifeng Dong, Michael Mortimore, Julian MC Golec, Peter Charlton

**Affiliations:** 1 Biology Department, Vertex Pharmaceuticals (Europe) Limited, 88 Milton Park, Abingdon, OX14 4RY, UK; 2 Department of Surgery, The Feinstein Institute for Medical Research, North Shore University Hospital and Long Island Jewish Medical Center, Manhasset, NY 11030, USA; 3 Chemistry Department, Vertex Pharmaceuticals (Europe) Limited, 88 Milton Park, Abingdon, OX14 4RY, UK

## Abstract

**Introduction:**

Prevention of lymphocyte apoptosis by caspase inhibition has been proposed as a novel treatment approach in sepsis. However, it has not been clearly demonstrated that caspase inhibitors improve survival in sepsis models when dosed post-insult. Also, there are concerns that caspase inhibitors might suppress the immune response. Here we characterize VX-166, a broad caspase inhibitor, as a novel potential treatment for sepsis.

**Methods:**

VX-166 was studied in a number of enzymatic and cellular assays. The compound was then tested in a murine model of endotoxic shock (lipopolysaccharide (LPS), 20 mg/kg IV) and a 10 d rat model of polymicrobial sepsis by caecal ligation and puncture (CLP).

**Results:**

VX-166 showed potent anti-apoptotic activity in vitro and inhibited the release of interleukin (IL)-1beta and IL-18. In the LPS model, VX-166 administered 0, 4, 8 and 12 h post-LPS significantly improved survival in a dose-dependent fashion (*P *< 0.0028). In the CLP model, VX-166 continuously administered by mini-osmotic pump significantly improved survival when dosed 3 h after insult, (40% to 92%, *P *= 0.009). When dosed 8 h post-CLP, VX-166 improved survival from 40% to 66% (*P *= 0.19). Mode of action studies in the CLP model confirmed that VX-166 significantly inhibited thymic atrophy and lymphocyte apoptosis as determined by flow cytometry (*P *< 0.01). VX-166 reduced plasma endotoxin levels (*P *< 0.05), suggesting an improved clearance of bacteria from the bloodstream. Release of IL-1beta in vivo or T-cell activation in vitro were moderately affected.

**Conclusions:**

Our studies enhance the case for the use of caspase inhibitors in sepsis. VX-166 itself has promise as a therapy for the treatment of sepsis in man.

## Introduction

Sepsis is a significant health problem and a major cause of death in intensive care units worldwide. Approximately 750,000 people are afflicted annually in the United States alone and, despite progress in intensive medical care, the mortality rate still ranges from 30 to 60% [1]. Novel treatment approaches, for example anti-cytokine therapies, have shown very limited success and the only approved drug Xigris (activated protein C) shows a minor improvement in survival in a restricted patient population [2-4]. Effective therapies are therefore desperately needed to reduce the morbidity and mortality associated with this disease.

Inhibition of apoptosis has recently been suggested as a novel therapeutic approach for the prevention and treatment of sepsis [5,6]. Studies in septic patients and animals have demonstrated excessive apoptosis, mainly in the intestine, lymphoid organs and circulating lymphocytes [7-10]. This loss of immune cells is believed to contribute to immune suppression that is linked to disease pathogenesis and the resultant mortality. The decrease in lymphocyte counts can occur within a day of disease onset, and patients dying from sepsis have increased numbers of apoptotic lymphocytes in both the peripheral circulation and the spleen [11-15]. Indeed, recent reports suggest a causative link between the profound lymphocyte loss due to apoptosis and poor outcome [15,16]. The rationale to pursue an inhibitor of apoptosis for the treatment of sepsis is further strengthened by studies where anti-apoptotic antibodies, small molecules or genetic approaches have led to a survival benefit in animal models. Importantly, it was found that prevention of lymphocyte apoptosis has a profound positive effect on survival in sepsis models [17-20].

Caspases represent important targets for the development of anti-apoptotic drugs. Members of the caspase family of proteases are essential for both the initiation and progression of apoptosis [21]. Caspases are upregulated in the lymphocytes of sepsis patients and are believed to facilitate lymphocyte death [22,23]. Both selective and broad caspase inhibitors have been reported to improve survival in sepsis models, and inhibition of lymphocyte apoptosis has been suggested as the key mode of action of these compounds [20,24-27].

Despite these promising results, questions remain regarding potential limitations of anti-apoptotic therapy in sepsis. In most published animal studies the caspase inhibitors were dosed at or shortly after the time of insult, thus reflecting prevention of septicaemia rather than the treatment of sepsis itself. This, however, does not validate caspase inhibition as a potential therapy for patients that present with the disease.

In addition there are still concerns about how effective inhibition of caspases will be in the clinical setting. For instance, caspase-8 has been reported to play an important role in T-cell activation and the adaptive immune system [28]. Thus, inhibition of caspase-8 could cause T-cell anergy and add to immune-suppression in sepsis rather than helping prevent it. Also a subset of caspases, in particular caspase-1, plays a key role in the activation of the pro-inflammatory cytokines IL-1β and IL-18 [29]. The impact of a potential anti-inflammatory component through inhibition of caspase-1 is difficult to predict in the context of sepsis. For example, elevated levels of IL-1β and IL-18 have been linked to the pathology of sepsis and endotoxic shock [30], and inhibition of these cytokines has been proposed as a potential therapeutic approach. However, anti-IL-1β therapy has proved to be ineffective in sepsis trials [31], and inhibition of caspase-1 has been suggested as potentially rendering individuals more susceptible to infections and severe sepsis [32].

**Figure 1:**
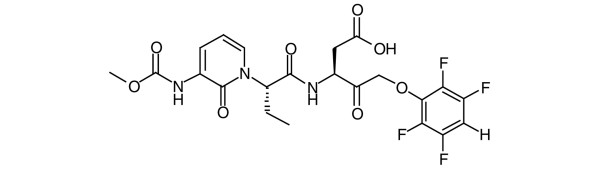
Structure of the broad caspase inhibitor VX-166.

In this report we characterise VX-166 (Figure 1), a broad caspase inhibitor with potent anti-apoptotic activity, as a novel potential treatment for sepsis. Importantly, we demonstrate that VX-166 improves survival in a caecal ligation and puncture (CLP) model when given several hours post insult. Furthermore, we alleviate some key issues regarding the immune suppressive properties of caspase inhibitors in the context of sepsis. Overall these data significantly strengthen the case for caspase inhibition in this disease. They also demonstrate that VX-166 itself represents significant progress in the development of therapeutically viable broad caspase inhibitors for the treatment of sepsis.

## Materials and methods

### Enzyme inhibition assays

Active recombinant caspase-1, -3, -4, -7 and -8 were prepared as described previously [33,34]. Caspase-2 was purchased from Biomol (Exeter, UK), caspase-6 from BD Biosciences (Oxford, UK), caspase-9 from Europa Bioproducts Ltd. (Cambridge, UK) and caspase-10 from Chemicon International Inc. (Harrow, UK). Cathepsin B and granzyme B were purchased from Merck Biosciences Ltd. (Nottingham, UK). Fluorogenic protease substrates were purchased from Bachem Ltd. (St. Helens, UK). All reactions were carried out at 37°C using a Molecular Devices (Wokingham, UK) Gemini plate-reader at 460 nm (excitation wavelength of 390 nm). Caspase inhibition assays were conducted, with minor modifications, as described by Margolin and colleagues [34]. Irreversible kinetics were analysed by calculations of second-order inactivation rate constants (k) using GraphPad (La Jolla, CA, USA) Prism 4.0 software. A larger number for an inactivation rate constant indicates a greater degree of inactivation. To determine the inhibitory effect on cathepsin B, 4.25 nM of the enzyme were added to 250 μM z-RR-AMC in 10 mM 2-morpholino-ethanesulfonic acid pH 6.0, 1 mM DTT, 1 mM EDTA and 0.03% Brij35 in the presence or absence of VX-166 and the reaction monitored continuously over 15 minutes. Granzyme B was assayed, with minor modifications, as described [35] by Thornberry and colleagues. Additional counterscreening against a panel of 83 additional enzymes, ion channels and receptors was carried out at MDS Pharma Services (Bothell, WA, USA).

### *In vitro *apoptosis assays in Jurkat cells

Jurkat cells were obtained from LGC Standards (Teddington, UK) and were grown in RPMI 1640, 10% FCS, 2 mM L-glutamine, 100 U/ml penicillin and 0.1 mg/ml streptomycin (all from Sigma Aldrich Ltd., Poole, UK) at 37°C in 5% CO_2_/95% humidity. Receptor- or stress-mediated apoptosis (2 × 10^5 ^cells/ml) was induced by addition of either anti-Fas antibody CH-11 (10 ng/ml, Millipore UK Ltd., Watford, UK), TNF-α (20 ng/ml, R&D Systems, Abingdon, UK) with cycloheximide (112 ng/ml, Sigma Aldrich Ltd., Poole, UK) or staurosporine (350 ng/ml, Sigma Aldrich Ltd., Poole, UK). VX-166 was diluted into DMSO (10 mM), further diluted in media and added at a range of concentrations. Cells were then incubated for 18 hours. Apoptotic cells were then labelled with Cy5-conjugated Annexin-V (BD Biosciences, Oxford, UK) and detected using a FMAT™ 8100 Fluorimetric Microassay Technology Reader (Applied Biosystems, Warrington, UK). For the experiments using anti-Fas antibody, apoptosis was additionally measured using a Cell Death Detection ELISA kit (Roche Diagnostics Ltd., Lewes, UK) following the manufacturer's instructions.

### *In vitro *apoptosis assay in human aorta endothelial cells

Human aorta endothelial cells (HAEC) were purchased from Cambrex Bio Science Wokingham Ltd., (Wokingham, UK) and cultured as recommended by the supplier. Apoptosis was induced in 5 × 10^4 ^cells/ml by addition of medium deprived of serum and growth factors. VX-166 was added at a range of concentrations as described above and the cells incubated for 48 hours at 37°C in an atmosphere of 5% CO_2_/95% humidity. Apoptosis was measured using a Cell Death Detection ELISA kit (Roche Diagnostics Ltd., Lewes, UK) following the manufacturer's instructions.

### *In vitro *IL-1β and IL-18 release assay from peripheral blood mononuclear cells

Peripheral blood mononuclear cells (PBMC) from healthy donors were isolated by Ficoll-Paque gradient centrifugation from buffy coats obtained from the National Blood Transfusion Service (Bristol, UK). For IL-1β release, 5 × 10^5 ^cells/ml were then stimulated with 50 ng/ml lipopolysaccharide (LPS) from Escherichia coli (Sigma-Aldrich Ltd., Poole, UK) in the presence or absence of VX-166 and incubated for 18 hours. Secretion of IL-1β into the supernatant was determined by ELISA (R&D Systems, Abingdon, UK) following the manufacturer's instructions. For IL-18 release, 7.5 × 10^5 ^cells/ml were stimulated with 6.25 × 10^-4^% (w/v) Staphylococcus aureus cell suspension (SAC; Sigma-Aldrich Ltd., Poole, UK) in the presence or absence of VX-166 and incubated overnight. The IL-18 content in the supernatants was quantified by ELISA (Caltag-MedSystems Ltd., Buckingham, UK) following the manufacturer's instructions. In both assays PBMC viability was assessed after the 18 hours incubation time using MTS assay (Promega UK Ltd., Southampton, UK) to ensure that the reduction in cytokine release was not caused by a cytotoxic effect of the compound.

### *In vitro *IL-2 release assay from peripheral blood mononuclear cells

PBMC from healthy donors were isolated as described above. To the wells of a 96-well plate previously coated with 1.5 μg/ml anti-hCD3 (Serotec, Kidlington, UK), 5 × 10^5 ^cells/ml and 1 μg/ml anti-hCD28 (BD Biosciences, Oxford, UK) were added. Plates were then incubated for 24 hours in the presence or absence of VX-166 or the calcineurin inhibitor FK506 (Merck Biosciences Ltd., Nottingham, UK). IL-2 release into the supernatant was subsequently determined by ELISA (R&D Systems, Abingdon, UK) following the manufacturer's instructions.

### Endotoxic shock in the mouse

Endotoxic shock studies were approved by the Vertex Pharmaceuticals Institutional Animal Care and Use Committee. Adult male CD-1 mice (weighing 30 to 32 g; Charles River Laboratories, Wilmington, MA, USA) were administered LPS (from *E. coli *serotype 0111:B4, Sigma-Aldrich, St. Louis, MO, USA) at 20 mg/kg iv and survival was monitored for 96 hours (n = 28 per group). VX-166 or vehicle (10% propylene glycol: 90% sodium phosphate buffer pH 8) were administered by repeat iv bolus at 0 hours (co-injection with LPS), 4, 8 and 12 hours post-LPS. VX-166 was administered at 1, 3, 10 or 30 mg/kg.

### Caecal ligation and puncture in the rat

CLP studies were performed in accordance with the National Institutes of Health guidelines for the use of experimental animals and were approved by the Institutional Animal Care and Use Committee of the Feinstein Institute for Medical Research. Adult male Sprague-Dawley rats (weighing 275 to 325 g, Charles River Laboratories, Wilmington, MA, USA) were fasted overnight then anaesthetised with isoflurane. The caecum was exposed via a 2 cm midline incision. It was ligated just distal to the ileocaecal valve, then punctured twice with an 18-gauge needle and gently squeezed to release a small amount of faecal material to ensure patency of the perforation sites before the abdominal incision was closed. Sham-operated rats underwent the same procedure with the exception that the caecum was neither ligated nor punctured. Animals received subcutaneous fluids (30 ml/kg saline) immediately after the procedure. No antibiotics were administered. In the 7 and 10 day survival studies animals were re-anaesthetised 20 hours after CLP, the perforated caecum was removed and the abdominal cavity washed twice with 40 ml of warm, sterilised saline. The abdominal cavity was closed, anaesthetics withdrawn and rats returned to their cages where they were allowed free access to food and water. In the 20 hour endpoint study the caecum was not removed. In this study blood samples were collected by cardiac puncture under terminal anaesthesia into heparinised syringes for the subsequent measurement of IL-1β and bacterial endotoxin, while the thymus was removed for analysis of thymocyte apoptosis.

Alzet^® ^mini-osmotic pumps (Durect Corporation, Cupertino, CA, USA) were used for continuous delivery of VX-166 or vehicle (25% dimethyl sulphoxide: 75% propylene glycol). Pumps with a 2 ml reservoir capacity and a 10 μl//h delivery rate were used in all studies. These pumps are designed to deliver for seven days and so would have expired two to three days before the end of the 10 day survival studies. The mini-osmotic pumps were filled with VX-166 (5 to 25 mg/ml or vehicle) and primed overnight at 37°C prior to subcutaneous implantation in anaesthetised rats. The 25 mg/ml concentration of VX-166 equates to an approximate dose of 0.83 mg/kg/h for a 300 g rat. The pumps were implanted immediately following CLP, except in the delayed onset of treatment study in which pumps were implanted either immediately following CLP (0 hour) or 3 or 8 hours following CLP. Sham-operated rats were implanted with vehicle filled mini-osmotic pumps.

### Determination of blood levels of IL-1β and endotoxin in the rat

Blood samples were centrifuged at 1500 g for 15 minutes at 4°C, the plasma was removed and stored at -80°C until analysis. Plasma IL-1β levels were determined by ELISA (R&D Systems Inc., Minneapolis, MN, USA) following the manufacturer's instructions. Plasma endotoxin levels were determined using a *Limulus *amebocyte lysate (LAL) kit (Associates of Cape Cod, Inc., East Falmouth, MA, USA) according to the manufacturer's instruction. Briefly, 50 μl plasma samples were diluted 1:10 in LAL reagent solution. Endotoxin (*E. coli *0113:H10, provided by the manufacturer) was used as the standard. Both standard and samples were heated for 15 minutes at 75°C and were then added to a 96-well plate. Pyrochrome (50 μl) was added to each well, mixed on a plate shaker for 30 seconds, and incubated for 35 minutes at 37°C followed by the addition of 25 μl 50% acetic acid to stop the reaction. The optical density was determined at 405 nm using a spectrophotometer. The levels of endotoxin were then calculated and expressed as EU/ml.

### Determination of thymocyte apoptosis by flow cytometry

Rat PBMC were isolated from heparinised blood by Ficoll Paque (GE Healthcare Bio-Sciences Corp., Piscataway, NJ, USA) density gradient centrifugation and washed three times with PBS (Invitrogen, Carlsbad, CA, USA). Thymocytes and splenocytes were obtained by gentle grinding of organs between frosted glass slides and passing cells through a Cellector^® ^Tissue Sieve (Bellco Glass Inc., Vineland, NJ, USA). Red blood cells in the splenocyte suspension were lysed with ammonium chloride potassium buffer (0.15 M NH_4_Cl, 10 mM KHCO_3_, 0.1 mM Na_2_EDTA in H_2_O). Cells were then washed twice in PBS, counted and reconstituted in Ca^2+^-rich Annexin-V binding buffer (BD Biosciences, San Jose, CA, USA) at a concentration of 1 × 10^7 ^cells/ml. A 100 μl sample of the cell suspension was stained with 2.5 μl Annexin-V-FITC and 1 μl propidium iodine (PI; BD Biosciences, San Jose, CA, USA) for 15 minutes and adjusted to a total volume of 500 μl with binding buffer. Cells were then analysed by flow cytometry with the FACS-Calibur (BD Biosciences, San Jose, CA, USA) and percentages of viable (Annexin-V^-^/PI^-^), early apoptotic (Annexin-V^+^/PI^-^), late apoptotic (Annexin-V^+^/PI^+^) and necrotic (Annexin-V^-^/PI^+^) cells were determined.

### Statistical analyses of *in vivo *studies

Survival data is shown as Kaplan-Meier survival curves with statistical analysis by the log-rank test. All other data is shown as mean ± standard error of the mean and statistical comparisons were by two-tailed unpaired Student's t-test or one-way analysis of variance followed by Dunnett's t-test where appropriate. Differences in values were considered significant if *P *< 0.05.

## Results

### VX-166 is a potent and selective broad caspase inhibitor

We screened VX-166 against a panel of recombinant caspases to confirm that the compound is a potent irreversible broad caspase inhibitor. VX-166 caused time-dependent inhibition of caspase activity with second order inactivation rate constants (k) ranging from 6 × 10^3 ^M/s against caspase-2 to more than 1 × 10^6 ^M/s against caspases -1 and -3 (Table 1).

**Table 1:** Second order inactivation rate constants (k) for VX-166 against a panel of nine caspases (n = 4 per assay).

Recombinant caspase	K (M/s) × 10^3^
Caspase-1	>1000 ^a^

Caspase-2	6.19 ± 0.51

Caspase-3	1171 ± 183

Caspase-4	480 ± 32

Caspase-6	77 ± 3.0

Caspase-7	526 ± 1.5

Caspase-8	194 ± 52

Caspase-9	131 ± 21

Caspase-10	49 ± 3.8

A larger number for an inactivation rate constant k indicates a greater degree of inactivation.

^a ^inactivation rate was above the upper limit of the assay.

To evaluate selectivity, VX-166 was counter-screened against the serine protease granzyme B and the cysteine protease cathepsin B. Granzyme B is a protease with similar substrate specificity to caspases [35] while cathepsin B is strongly inhibited by a number of commercially available caspase inhibitors [36,37]. VX-166 did not show any inhibitory activity against granzyme B at 50 μM and only weak inhibition of cathepsin B at the highest concentration tested (19% inhibition at 50 μM). VX-166 (5 μM) was tested against a panel of 83 additional enzymes, ion channels and receptors that included 28 proteases. No significant cross-reactivity was seen (data not shown). These data indicate that VX-166 is highly selective for caspases.

### VX-166 prevents apoptosis in vitro

We performed a number of cellular assays to demonstrate that VX-166 has the ability to inhibit apoptotic processes that are relevant in sepsis. Programmed cell death of T-cells in sepsis is mediated by a number of stimuli (e.g. Fas ligand, TNF-α or steroids) and can proceed via different apoptotic pathways [6,22]. We therefore tested VX-166 in cell assays with a variety of apoptotic stimuli using the Jurkat T-cell lymphoma line. As shown in Table 2, VX-166 potently inhibits apoptosis in Jurkat cells induced by both receptor-driven (Fas, TNFR) and stress-driven (staurosporine) caspase activation pathways. Fas-induced apoptosis was particularly sensitive to inhibition by VX-166 as determined by Annexin-V staining and DNA-fragmentation.

**Table 2:** Inhibition of apoptosis by VX-166 in a variety of cell-based assays (n = 4 per assay).

Apoptotic stimulus	Cell type/readout	IC_50 _(nM)
Anti-Fas	Human Jurkat cell line/Annexin V	27 ± 9

Anti-Fas	Human Jurkat cell line/DNA fragmentation	45 ± 7

TNF-α/cycloheximide	Human Jurkat cell line/Annexin V	120 ± 50

Staurosporine	Human Jurkat cell line/Annexin V	255 ± 155

Serum withdrawal and nutrient deprivation	HAEC/DNA fragmentation	310 ± 70

IC_50 _= 50% inhibitory concentration; HAEC = human aorta endothelial cells.

Endothelial cell apoptosis has recently also been suggested to occur in sepsis [38,39], potentially contributing to vascular damage causing leakage of fluid into the extravascular space and collapse of the microcirculation. We therefore investigated the ability of VX-166 to prevent the death of human primary endothelial cells *in vitro*. VX-166 inhibited endothelial cell death potently, with a 50% inhibitory concentration (IC_50_) value of 310 nM (Table 2). In summary our data suggest that VX-166 rescues human cells from apoptosis regardless of apoptotic stimulus or cell type.

### VX-166 inhibits the release of pro-inflammatory cytokines from human primary cells

To confirm that an inhibitor of caspases -1 and -4 inhibited the release of IL-1β and IL-18, we tested the effect of VX-166 on cytokine-release from endotoxin-treated PBMC. VX-166 potently inhibited the release of both IL-1β and IL-18 with IC_50 _values below 500 nM (Table 3). A viability assay conducted simultaneously confirmed that this inhibition of cytokine release was not due to a cytotoxic effect of the compound.

**Table 3:** Inhibition of cytokine release by VX-166 (n = 4 per assay).

Cytokine/cell type	IC50 ± SD (nM)
IL-1β/LPS-treated human PBMC	260 ± 120

IL-18/SAC-treated human PBMC	450 ± 170

IC_50 _= 50% inhibitory concentration; LPS = lipopolysaccharide; PBMC = peripheral blood mononuclear cells; SAC = *Staphylococcus aureus *cell suspension; SD = standard deviation.

### VX-166 significantly improves survival in a murine model of endotoxic shock

**Figure 2:**
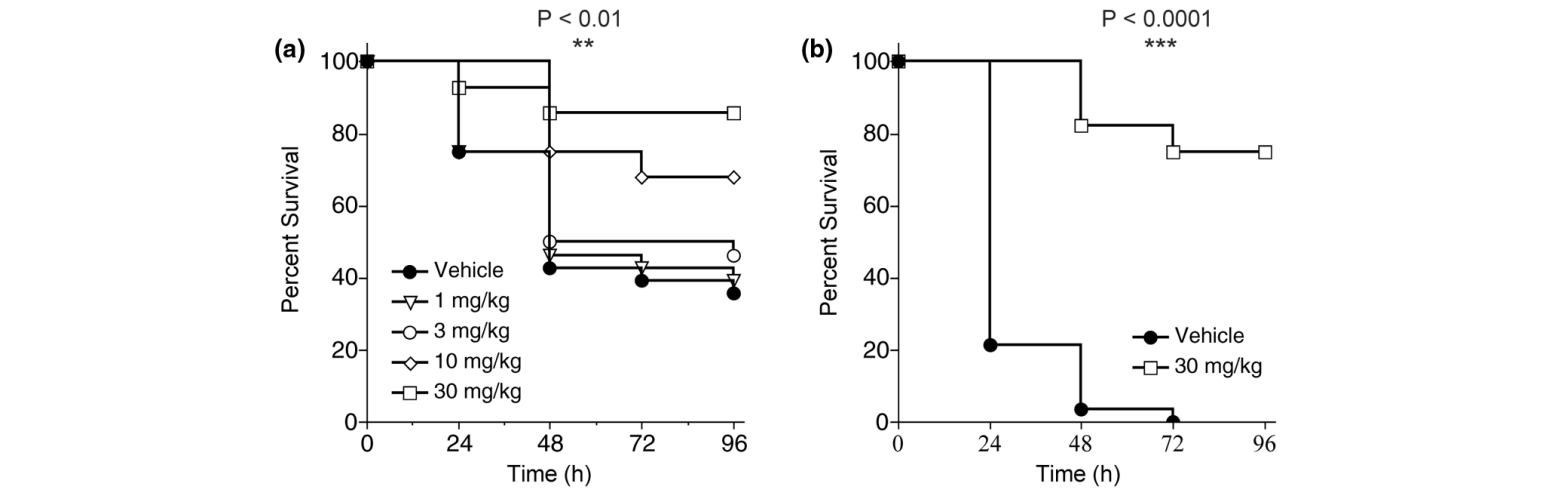
VX-166 significantly improves survival in an endotoxic shock model in the mouse.  Survival was monitored for 96 hours following administration of a lethal dose of lipopolysaccharide (LPS; 20 mg/kg iv) to male CD-1 mice (n = 28 per group). VX-166 was administered by repeat iv bolus at 0, 4, 8 and 12 hours following LPS. **(a) **Dose response of VX-166 dosed at 1 (triangles), 3 (empty circles), 10 (diamonds) or 30 mg/kg (squares) vs. vehicle control (full circles); *P < 0.01. **(b) **Single dose repeat study using the optimal dose (30 mg/kg) of VX-166 (squares) vs. vehicle control (circles); *P < 0.001. Survival in the vehicle group was different from **(a) **experiment due to a different batch of LPS used.

The attractive *in vitro *profile of VX-166 next led us to test the compound in a rapid and well-established murine model of endotoxic shock. Male CD-1 mice (n = 28 per group) were administered LPS (20 mg/kg iv) and survival was monitored for 96 hours. VX-166 administered by repeat iv bolus (0, 4, 8 and 12 hours post-LPS) significantly improved survival in a dose-dependent fashion (Figure 2a; log-rank test *P *< 0.01). The highest dose (30 mg/kg) more than doubled the survival rate at 96 hours compared with vehicle control. The protective effect of VX-166 was confirmed in a second study using the optimal dose of 30 mg/kg. VX-166 again substantially improved survival compared with vehicle control despite an increase in mortality in the control group due to a different batch of LPS used (Figure 2b; *P *< 0.0001). Surviving mice from the VX-166 (30 mg/kg) treated group fully recovered from the endotoxaemia and regained normal behaviour patterns. Thus VX-166 can prevent death due to endotoxic shock rather than merely delay it.

### VX-166 significantly improves survival in the rat CLP model

**Figure 3:**
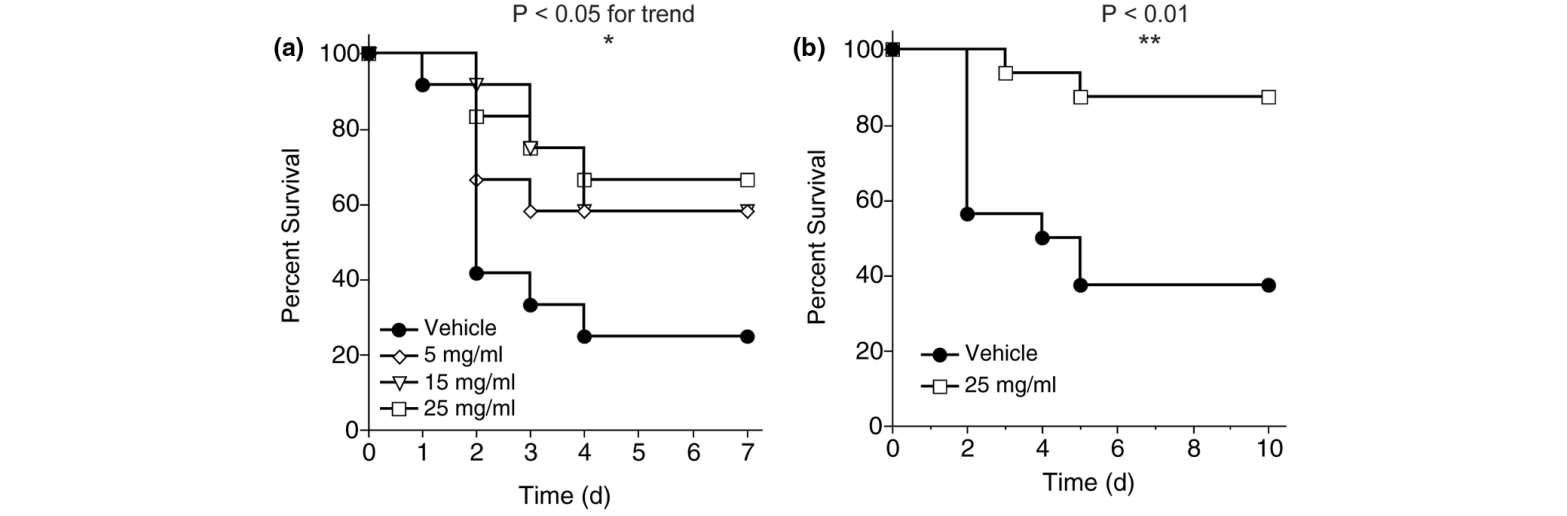
VX-166 significantly improves survival following CLP in the rat.  **(a) **Dose response of VX-166. The compound was administered by mini-osmotic pump (10 μl/h for seven days) implanted subcutaneously immediately after caecal ligation and puncture (CLP) and survival was monitored over seven days(n = 12 per group). VX-166 was dosed at 5 (diamonds), 15 (triangles), or 25 mg/ml (squares) vs. vehicle control (circles); *P < 0.05 logrank test for trend. **(b) **Single dose study using the optimal dose (25 mg/ml) of VX-166 (squares) vs. vehicle control (circles). The compound was administered by mini-osmotic pump (10 μl/h for seven days) implanted subcutaneously immediately after CLP and survival was monitored over 10 days (n = 16 per group); *P < 0.01.

VX-166 was assessed in a CLP model in the rat. This particular model is one of the most relevant models of sepsis and mimics the human diseases of ruptured appendicitis or perforated diverticulitis [40]. In two studies, VX-166 was continuously administered by mini-osmotic pump implanted immediately following CLP. Because of the higher relevance of the model continuous infusion was chosen to reflect the potential dosing regime in the clinic. In the first study (Figure 3a; n = 12 per group) survival was monitored for seven days. VX-166 was of significant benefit (*P *< 0.05 log-rank test for trend), increasing survival at day 7 from 25% in the control group to 67% with VX-166 at 25 mg/ml. The efficacy of the optimal dose of VX-166 (25 mg/ml) was confirmed in the second study (Figure 3b, n = 16 per group) in which the observation period was extended to 10 days. Again VX-166 significantly (*P *< 0.01) improved survival compared with vehicle control.

### VX-166 significantly improves survival in the rat CLP model when dosed post insult

**Figure 4:**
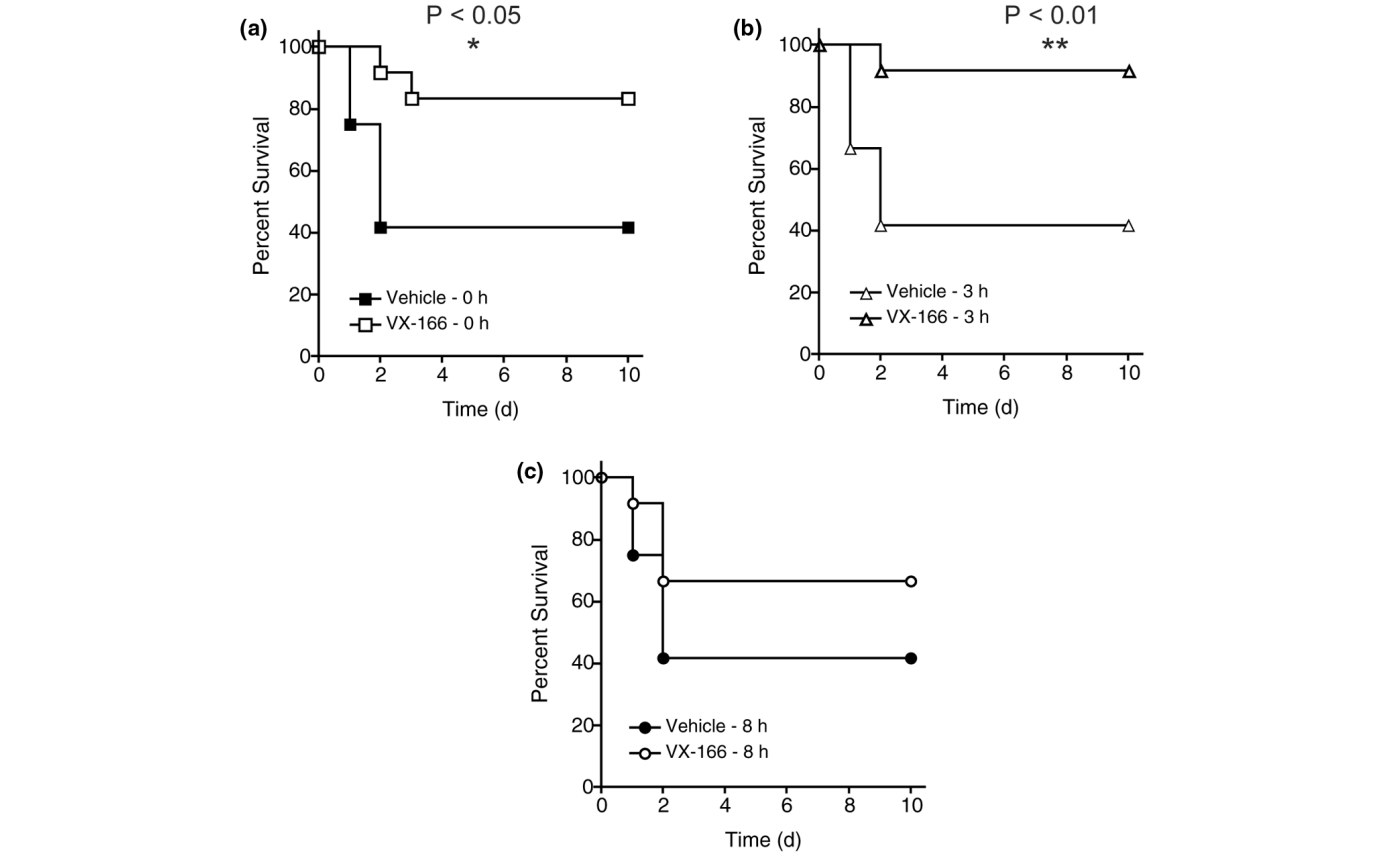
VX-166 improves survival following CLP in the rat when dosed post insult.  The compound was administered by mini-osmotic pump (10 μl/h for seven days, 25 mg/ml) implanted subcutaneously at different times after caecal ligation and puncture (CLP) and survival was monitored over 10 days (n = 12 per group). **(a) **VX-166 dosed at 0 hours (empty squares) vs. vehicle control (filled squares); *P = 0.026 **(b) **VX-166 dosed at three hours (empty triangles) vs. vehicle control (filled triangles); *P = 0.009. **(c) **VX-166 dosed at eight hours (empty circles) vs. vehicle control (filled circles).

Previous reports have shown that caspase inhibitors can improve survival in sepsis models when dosed near the time of insult [20,24-27]. However, the effect of delaying the administration of a caspase inhibitor in the CLP model has not been fully explored. Therefore, we assessed the therapeutic potential of VX-166 by investigating the effect of delaying treatment until after CLP (n = 12 per group). Previous reports have shown that CLP rats suffer from sepsis within two to five hours post insult [41-43]. VX-166 (25 mg/ml) was administered by mini-osmotic pump starting 0, 3 or 8 hours after CLP. Survival was monitored for 10 days and compared with a separate vehicle control group for each time point. As expected, VX-166 significantly increased survival at day 10 to 83% compared with 40% in the control group (*P *= 0.026, Figure 4a) when dosed simultaneously with CLP. When dosing was initiated three hours after insult, VX-166 still retained its efficacy and significantly improved survival from 40% to 92% (*P *= 0.009, Figure 4b). When initiation of treatment was delayed further, eight hours post-CLP, VX-166 still improved survival from 40% to 66%; this result did not quite reach statistical significance (*P *= 0.19, Figure 4c). It should be noted that at this time point the peripheral circulation is already severely affected in CLP rats, potentially leading to an impairment of compound delivery from the mini-osmotic pumps. Our results therefore indicate that caspase inhibitors such as VX-166 may be useful as a sepsis therapy.

### VX-166 preserves lymphocytes, lowers endotoxin levels and dampens the proinflammatory cytokine response following CLP in the rat

**Figure 5:**
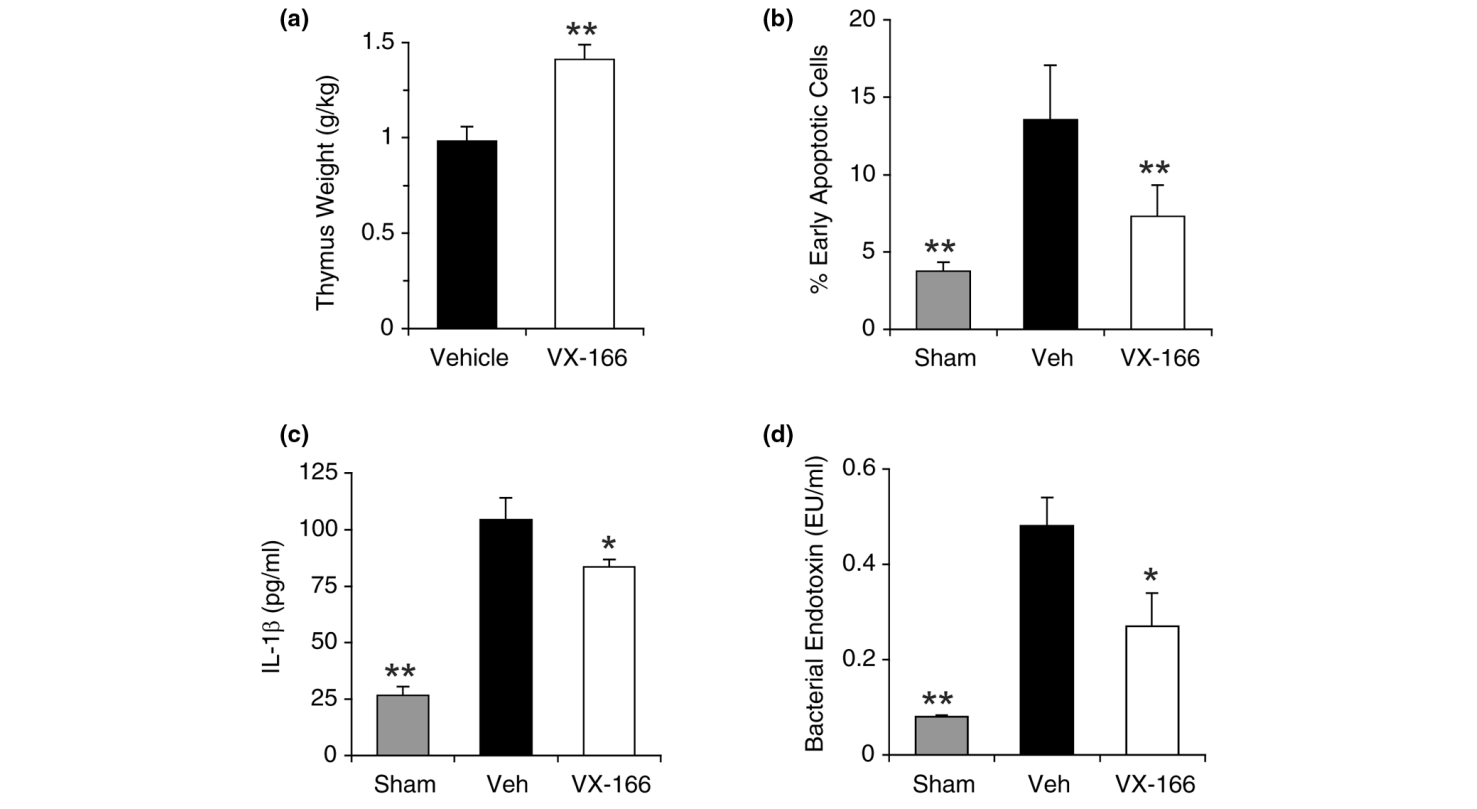
VX-166 has a beneficial effect on lymphocyte loss, endotoxin and IL-1β levels in CLP.  **(a) **Thymocyte weight of caecal ligation and puncture (CLP) rats from the single dose 10 days survival study was determined in surviving animals at day 10. VX-166 was dosed at 25 mg/ml (white bar) vs. vehicle control (black bar); *P < 0.01. **(b) **Lymphocyte apoptosis was determined 20 hours post CLP (n = 10 per group) in VX-166-treated (25 mg/ml, white bars) vs. vehicle treated CLP rats (black bar) and sham controls (grey bar); *P < 0.01 vs. vehicle control. **(c) **Plasma IL-1β levels were determined 20 hours post CLP (n = 10 per group) in VX-166-treated (25 mg/ml, white bars) vs. vehicle treated CLP rats (black bar) and sham controls (grey bar); *P < 0.05 vs. vehicle control. **(d) **Plasma endotoxin levels were determined 20 hours post CLP (n = 10 per group) in VX-166-treated (25 mg/ml, white bars) vs. vehicle treated CLP rats (black bar) and sham controls (grey bar); *P < 0.05 vs. vehicle control.

Previous studies have suggested that inhibition of lymphocyte apoptosis is one of the key mechanisms through which caspase inhibition provides protection in septic animals [18]. In experimental sepsis thymocyte apoptosis is associated with rapid thymic weight loss that can be used as a simple marker of cell death [33,34]. In the second CLP study (Figure 3b) we measured thymus weight from surviving rats on day 10. We found a significantly higher weight (*P *< 0.01) for VX-166-treated rats compared with control animals (Figure 5a), indicating the inhibition of thymocyte death *in vivo*. These results are likely to be an underestimate of the protective effect of VX-166 on thymus weight because only those control rats still surviving on day 10 were included in the analysis. The effect of VX-166 on lymphocyte apoptosis was further investigated in a CLP study utilising an earlier (20 hour) end point when all the control animals were still alive. VX-166 (25 mg/ml) treated CLP rats were compared with vehicle CLP rats and a third group of sham-operated animals that also received vehicle mini-osmotic pumps (n = 10 per group). The proportion of thymocytes undergoing apoptosis or necrosis at 20 hours was determined by flow cytometry. The CLP procedure induced a significant increase in early apoptotic thymocytes in the vehicle CLP group compared with sham control (Figure 5b), and a corresponding reduction in the percentage of Annexin-V^-^/PI^- ^viable cells. VX-166 significantly (*P *< 0.01) reduced the percentage of early apoptotic thymocytes compared with the vehicle CLP group and, as a consequence, significantly increased the percentage of viable cells. The level of necrotic cells remained unchanged between the different groups. Thus inhibition of lymphocyte apoptosis appears to be one of the mechanisms by which VX-166 increases survival following CLP.

To investigate the effect of VX-166 on the inflammatory response, blood samples were taken at the 20 hour time point to measure the circulating levels of IL-1β and bacterial endotoxin. At 20 hours, IL-1β levels were strongly increased in the CLP vehicle group over sham operated but VX-166 caused a significant reduction in the level of the cytokine (*P *< 0.05, Figure 5c) compared with vehicle alone. However, the IL-1β levels in VX-166-treated animals remained elevated compared with sham controls. VX-166 also significantly (*P *< 0.05) reduced the level of endotoxin in the blood compared with the vehicle CLP group (Figure 5d), which may be indicative of improved clearance mechanisms.

### VX-166 does not inhibit the activation of human T-cells *in vitro *below 100 μM

**Figure 6:**
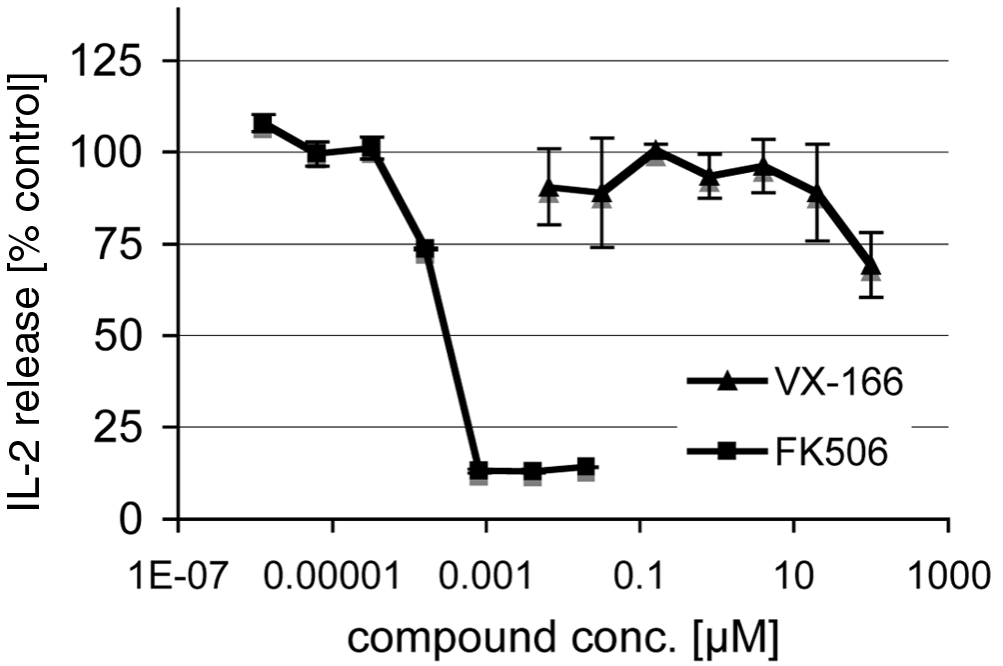
VX-166 does not inhibit the activation of human T-cells at concentrations below 100 μM.  VX-166 and the calcineurin-inhibitor FK506 were tested in an IL-2 assay using anti-CD28/anti-CD3 stimulated human peripheral blood mononuclear cells. The compounds were added directly after stimulation and incubated for 24 hours. IL-2 secretion into the supernatant was determined by ELISA. FK506 inhibited the release of IL-2 with a 50% inhibitory concentration (IC_50_) value of 0.2 nM while VX-166 partly inhibited the release of IL-2 by ca. 25% at 100 μM.

Several reports in the literature have suggested that caspases are not only involved in cytokine release and apoptosis but also in the activation of lymphocytes. Caspase-8-deficient primary human T-lymphocytes have been reported to be defective in IL-2-production after T-cell receptor stimulation [28]. Despite the protection of rodent T-cells in the CLP model a possibility remained that VX-166 may cause anergy in stimulated human lymphocytes. We therefore tested VX-166 in an IL-2 release assay using anti-CD3/CD28 stimulated human PBMC together with the calcineurin inhibitor FK506 as a positive control (Figure 6). FK506 inhibited IL-2 release potently with an IC_50 _value of 0.2 nM, matching values published by others [43]. VX-166 reduced IL-2 release by only 25% at 100 μM, a concentration well above the level at with VX-166 completely inhibits cellular apoptosis. The concentration of 100 μM also far exceeds the plasma levels obtained with VX-166 *in vivo *(data not shown). This result suggests that VX-166 is unlikely to affect the activation of human T-cells *in vivo*, which is in line with the observed preservation of the immune response in the CLP model.

## Discussion

Our studies with VX-166 confirm other reports that a potent small molecule caspase inhibitor can be utilised to achieve a significant survival benefit in animal models of endotoxic shock and polymicrobial sepsis [20,24,44,45]. We also observe inhibition of lymphocyte apoptosis *in vivo *in line with reports by Hotchkiss and colleagues [20,24] and Oberholzer and colleagues [27]. Most importantly we demonstrate that VX-166 still improves survival when dosed to CLP rats that are already suffering from sepsis [41,42]. This shows for the first time that caspase inhibition is able to improve survival in septic animals when used therapeutically. VX-166 loses some of its efficacy when dosed eight hours post insult, a timepoint where lymphocyte apoptosis is already evident in this model (P. Wang, unpublished). This may indicate that a caspase inhibitor is best used before the onset of lymphocyte death in cases of sepsis. Alternatively the compound delivery by mini-osmotic pump may have been impaired due to dehydration and circulation shutdown of the animals, preventing a better outcome.

Although we determined the impact of VX-166 on T-cells it cannot be ruled out that the compound may have affected other leukocytes, e.g. neutrophils. The half-life of neutrophils is increased in sepsis due to delayed apoptosis, and increased neutrophil apoptosis has been suggested to aid the resolution of the inflammatory process in sepsis [46]. Further studies that assess the effect of VX-166 on neutrophils and include peripheral granulocyte as well as lymphocyte counts are therefore warranted.

One concern about the use of caspase inhibitors in sepsis, despite positive animal data, is the risk of causing T-cell anergy in humans. This arises from reports that caspase-8 is required for T-cell activation because mutations in the caspase-8 gene have been shown to cause suppression of the adaptive immune response in humans [28]. Furthermore, caspase-8 inhibition with 50 μM of the peptidic caspase inhibitors zVAD-FMK or IETD-FMK has been described to block IL-2 release from activated T-cells [47]. VX-166 causes partial inhibition on T-cell activation *in vitro *at 100 μM. However, this moderate effect needs to be compared with the nanomolar IC_50 _values obtained for this compound in apoptosis assays. In fact, zVAD-FMK gave an IC_50 _value of 230 nM in our Fas-assay (data not shown), indicating that there is a substantial window between the anti-apoptotic and T-cell-suppressive activity of caspase inhibitors in general. Our data therefore alleviate some concerns about the use of caspase inhibitors in sepsis patients.

VX-166 is also a potent inhibitor of inflammatory caspases, which translates into potent inhibition of cytokine release *in vitro*. When dosed in the CLP model VX-166 causes attenuation of the elevated IL-1β levels 20 hours post insult, but without complete suppression. This finding may be explained by a recent report that describes caspase-1-independent release of IL-1β from neutrophils [48]. Preliminary data using VX-166 as part of a racemic mixture indicate that the release of other cytokines such as TNF-α, IL-6 and IL-10 may also be affected by caspase inhibition (data not shown). VX-166 may therefore have a general dampening effect on cytokine release; however, this requires further investigation. VX-166 also reduces blood endotoxin levels in CLP. It is not clear whether this effect is due to a reduction of bacterial counts as described by Hotchkiss and colleagues [24] or an alteration of endotoxin clearance due to improved organ function. This question will be dealt with in future studies. Our data suggest that VX-166 is capable of dampening the release of IL-1β at concentrations where it causes strong inhibition of apoptosis and improvement in clearance of endotoxins from the blood stream. This profile is extremely appealing as recent data using TLR4 antagonists suggest that a modulation of pro-inflammatory cytokine release could be beneficial in sepsis [49].

## Conclusions

In summary we demonstrate that VX-166 is able to rescue septic animals from death and alleviate concerns about potential detrimental effects of broad caspase inhibitors in the context of sepsis. Our studies with VX-166 enhance the case for the use of caspase inhibitors in this indication. VX-166 itself has promise as an anti-apoptotic therapy for the treatment of sepsis in humans.

## Key messages

• VX-166, a broad caspase inhibitor, significantly improves survival in experimental sepsis when dosed post insult.

• Several concerns about the potential immune-suppressive properties of broad caspase inhibitors in the context of sepsis have been addressed.

• The studies with VX-166 enhance the case for the use of caspase inhibitors in sepsis.

## Abbreviations

CLP: caecal ligation and puncture; ELISA: enzyme-linked immunosorbent assay; FCS: fetal calf serum; HAEC: human aorta endothelial cells; IC_50_: 50% inhibitory concentration; IL: interleukin; k: second-order inactivation rate constants; LAL: *Limulus *amebocyte lysate; LPS: lipopolysaccharide; PBMC: peripheral blood mononuclear cells; PBS: phosphate-buffered saline; PI: propidium iodine; SAC: *Staphylococcus aureus *cell suspension.

## Competing interests

This study was sponsored by Vertex Pharmaceuticals (Europe) Ltd. and Vertex Pharmaceuticals Inc., the parent company of Vertex Pharmaceuticals (Europe) Ltd. Vertex Pharmaceuticals (Europe) Ltd. will also finance the manuscript. Peter Weber, Paul Wang, Michael Mortimore, Julian Golec and Peter Charlton are Vertex employees and hold stocks or stock options in Vertex Pharmaceuticals Inc. Stephane Maddens is a former Vertex employee. Ping Wang has previously acted as a consultant to Vertex. Vertex Pharmaceuticals Inc. holds patents or is applying for patents related to chemical matter that is the subject of the paper. Peter Weber, Michael Mortimore and Julian Golec are listed as inventors on some of these patents.

## Authors' contributions

PeWe oversaw the *in vitro *characterisation of VX-166, participated in the study design and drafted the manuscript. PiWa designed and coordinated the CLP studies and helped to draft the manuscript. SM performed the characterisation of VX-166 in cellular assays. PaWa carried out the enzymology studies. RW, MiMi and WD performed the CLP studies. MM coordinated the design of VX-166. JG and PC participated in the design of the study and helped to draft the manuscript. All authors read and approved the final manuscript.

## References

[B1] Awad SS State-of-the-art therapy for severe sepsis and multisystem organ dysfunction Am J Surg 2003 186 23S 30S 10.1016/j.amjsurg.2003.10.004 14684222

[B2] Dellinger RP Levy MM Carlet JM Bion J Parker MM Jaeschke R Reinhart K Angus DC Brun-Buisson C Beale R Calandra T Dhainaut JF Gerlach H Harvey M Marini JJ Marshall J Ranieri M Ramsay G Sevransky J Thompson BT Townsend S Vender JS Zimmerman JL Vincent JL Surviving Sepsis Campaign: international guidelines for management of severe sepsis and septic shock: 2008 Crit Care Med 2008 36 296 327 10.1097/01.CCM.0000298158.12101.41 18158437

[B3] Riedemann NC Guo RF Ward PA The enigma of sepsis J Clin Invest 2003 112 460 467 12925683 10.1172/JCI19523 PMC171398

[B4] Shimaoka M Park EJ Advances in understanding sepsis Eur J Anaesthesiol Suppl 2008 42 146 153 10.1017/S0265021507003389 18289433 PMC2490719

[B5] Hotchkiss RS Nicholson DW Apoptosis and caspases regulate death and inflammation in sepsis Nat Rev Immunol 2006 6 813 822 10.1038/nri1943 17039247

[B6] Wesche DE Lomas-Neira JL Perl M Chung CS Ayala A Leukocyte apoptosis and its significance in sepsis and shock J Leukoc Biol 2005 78 325 337 10.1189/jlb.0105017 15817707

[B7] Wang SD Huang KJ Lin YS Lei HY Sepsis-induced apoptosis of the thymocytes in mice J Immunol 1994 152 5014 5021 8176219

[B8] Hotchkiss RS Swanson PE Freeman BD Tinsley KW Cobb JP Matuschak GM Buchman TG Karl IE Apoptotic cell death in patients with sepsis, shock, and multiple organ dysfunction Crit Care Med 1999 27 1230 1251 10.1097/00003246-199907000-00002 10446814

[B9] Chung CS Wang W Chaudry IH Ayala A Increased apoptosis in lamina propria B cells during polymicrobial sepsis is FasL but not endotoxin mediated Am J Physiol Gastrointest Liver Physiol 2001 280 G812 G818 11292588 10.1152/ajpgi.2001.280.5.G812

[B10] Schroeder S Lindemann C Decker D Klaschik S Hering R Putensen C Hoeft A von Ruecker A Stüber F Increased susceptibility to apoptosis in circulating lymphocytes of critically ill patients Langenbecks Arch Surg 2001 386 42 46 10.1007/s004230000181 11405088

[B11] Roth G Moser B Krenn C Brunner M Haisjackl M Almer G Gerlitz S Wolner E Boltz-Nitulescu G Ankersmit HJ Susceptibility to programmed cell death in T-lymphocytes from septic patients: a mechanism for lymphopenia and Th2 predominance Biochem Biophys Res Commun 2003 308 840 846 10.1016/S0006-291X(03)01482-7 12927795

[B12] Ayala A Chung CS Xu YX Evans TA Redmond KM Chaudry IH Increased inducible apoptosis in CD4+ T lymphocytes during polymicrobial sepsis is mediated by Fas ligand and not endotoxin Immunology 1999 97 45 55 10.1046/j.1365-2567.1999.00765.x 10447713 PMC2326799

[B13] Hotchkiss RS Tinsley KW Swanson PE Schmieg RE Hui JJ Chang KC Osborne DF Freeman BD Cobb JP Buchman TG Karl IE Sepsis-induced apoptosis causes progressive profound depletion of B and CD4+ T lymphocytes in humans J Immunol 2001 166 6952 6963 11359857 10.4049/jimmunol.166.11.6952

[B14] Hotchkiss RS Tinsley KW Swanson PE Grayson MH Osborne DF Wagner TH Cobb JP Coopersmith C Karl IE Depletion of dendritic cells, but not macrophages, in patients with sepsis J Immunol 2002 168 2493 2500 11859143 10.4049/jimmunol.168.5.2493

[B15] Bilbault P Lavaux T Lahlou A Uring-Lambert B Gaub MP Ratomponirina C Meyer N Oudet P Schneider F Transient Bcl-2 gene down-expression in circulating mononuclear cells of severe sepsis patients who died despite appropriate intensive care Intensive Care Med 2004 30 408 415 10.1007/s00134-003-2118-z 14722631

[B16] Le Tulzo Y Pangault C Gacouin A Guilloux V Tribut O Amiot L Tattevin P Thomas R Fauchet R Drénou B Early circulating lymphocyte apoptosis in human septic shock is associated with poor outcome Shock 2002 18 487 494 10.1097/00024382-200212000-00001 12462554

[B17] Guo RF Huber-Lang M Wang X Sarma V Padgaonkar VA Craig RA Riedemann NC McClintock SD Hlaing T Shi MM Ward PA Protective effects of anti-C5a in sepsis-induced thymocyte apoptosis J Clin Invest 2000 106 1271 1280 10.1172/JCI10793 11086028 PMC381438

[B18] Hotchkiss RS Swanson PE Knudson CM Chang KC Cobb JP Osborne DF Zollner KM Buchman TG Korsmeyer SJ Karl IE Overexpression of Bcl-2 in transgenic mice decreases apoptosis and improves survival in sepsis J Immunol 1999 162 4148 4156 10201940

[B19] Iwata A Stevenson VM Minard A Tasch M Tupper J Lagasse E Weissman I Harlan JM Winn RK Over-expression of Bcl-2 provides protection in septic mice by a trans effect J Immunol 2003 171 3136 3141 12960340 10.4049/jimmunol.171.6.3136

[B20] Hotchkiss RS Tinsley KW Swanson PE Chang KC Cobb JP Buchman TG Korsmeyer SJ Karl IE Prevention of lymphocyte cell death in sepsis improves survival in mice Proc Natl Acad Sci USA 1999 96 14541 14546 10.1073/pnas.96.25.14541 10588741 PMC24472

[B21] Salvesen GS Riedl SJ Caspase mechanisms Adv Exp Med Biol 2008 615 13 23 full_text 18437889 10.1007/978-1-4020-6554-5_2

[B22] Hotchkiss RS Osmon SB Chang KC Wagner TH Coopersmith CM Karl IE Accelerated lymphocyte death in sepsis occurs by both the death receptor and mitochondrial pathways J Immunol 2005 174 5110 5118 15814742 10.4049/jimmunol.174.8.5110

[B23] Tinsley KW Cheng SL Buchman TG Chang KC Hui JJ Swanson PE Karl IE Hotchkiss RS Caspases -2, -3, -6, and -9, but not caspase-1, are activated in sepsis-induced thymocyte apoptosis Shock 2000 13 1 7 10.1097/00024382-200013010-00001 10638661

[B24] Hotchkiss RS Chang KC Swanson PE Tinsley KW Hui JJ Klender P Xanthoudakis S Roy S Black C Grimm E Aspiotis R Han Y Nicholson DW Karl IE Caspase inhibitors improve survival in sepsis: a critical role of the lymphocyte Nat Immunol 2000 1 496 501 10.1038/82741 11101871

[B25] Catalan MP Esteban J Subirá D Egido J Ortiz A Inhibition of caspases improves bacterial clearance in experimental peritonitis Perit Dial Int 2003 23 123 126 12713077

[B26] Méthot N Huang J Coulombe N Vaillancourt JP Rasper D Tam J Han Y Colucci J Zamboni R Xanthoudakis S Toulmond S Nicholson DW Roy S Differential efficacy of caspase inhibitors on apoptosis markers during sepsis in rats and implication for fractional inhibition requirements for therapeutics J Exp Med 2004 199 199 207 10.1084/jem.20031791 14718517 PMC2211770

[B27] Oberholzer C Tschoeke SK Moldawer LL Oberholzer A Local thymic caspase-9 inhibition improves survival during polymicrobial sepsis in mice J Mol Med 2006 84 389 395 10.1007/s00109-005-0017-1 16453149

[B28] Chun HJ Zheng L Ahmad M Wang J Speirs CK Siegel RM Dale JK Puck J Davis J Hall CG Skoda-Smith S Atkinson TP Straus SE Lenardo MJ Pleiotropic defects in lymphocyte activation caused by caspase-8 mutations lead to human immunodeficiency Nature 2002 419 395 399 10.1038/nature01063 12353035

[B29] Gu Y Kuida K Tsutsui H Ku G Hsiao K Fleming MA Hayashi N Higashino K Okamura H Nakanishi K Kurimoto M Tanimoto T Flavell RA Sato V Harding MW Livingston DJ Su MS Activation of interferon-gamma inducing factor mediated by interleukin-1 beta converting enzyme Science 1997 275 206 209 10.1126/science.275.5297.206 8999548

[B30] Elenkov IJ Iezzoni DG Daly A Harris AG Chrousos GP Cytokine dysregulation, inflammation and well-being Neuroimmunomodulation 2005 12 255 269 10.1159/000087104 16166805

[B31] Marshall JC Clinical trials of mediator-directed therapy in sepsis: what have we learned? Intensive Care Med 2000 26 Suppl 1 S75 S83 10.1007/s001340051122 10786962

[B32] Scott AM Saleh M The inflammatory caspases: guardians against infections and sepsis Cell Death Differ 2007 14 23 31 10.1038/sj.cdd.4402026 16977333

[B33] Wei Y Fox T Chambers SP Sintchak J Coll JT Golec JM Swenson L Wilson KP Charifson PS The structures of caspases-1, -3, -7 and -8 reveal the basis for substrate and inhibitor selectivity Chem Biol 2000 7 423 432 10.1016/S1074-5521(00)00123-X 10873833

[B34] Margolin N Raybuck SA Wilson KP Chen W Fox T Gu Y Livingston DJ Substrate and inhibitor specificity of interleukin-1 beta-converting enzyme and related caspases J Biol Chem 1997 272 7223 7228 10.1074/jbc.272.11.7223 9054418

[B35] Thornberry NA Rano TA Peterson EP Rasper DM Timkey T Garcia-Calvo M Houtzager VM Nordstrom PA Roy S Vaillancourt JP Chapman KT Nicholson DW A combinatorial approach defines specificities of members of the caspase family and granzyme B. Functional relationships established for key mediators of apoptosis J Biol Chem 1997 272 17907 17911 10.1074/jbc.272.29.17907 9218414

[B36] Schotte P Declercq W Van HS Vandenabeele P Beyaert R Non-specific effects of methyl ketone peptide inhibitors of caspases FEBS Lett 1999 442 117 121 10.1016/S0014-5793(98)01640-8 9923616

[B37] Rozman-Pungercar J Kopitar-Jerala N Bogyo M Turk D Vasiljeva O Stefe I Vandenabeele P Brömme D Puizdar V Fonović M Trstenjak-Prebanda M Dolenc I Turk V Turk B Inhibition of papain-like cysteine proteases and legumain by caspase-specific inhibitors: when reaction mechanism is more important than specificity Cell Death Differ 2003 10 881 888 10.1038/sj.cdd.4401247 12867995

[B38] Matsuda N Hattori Y Vascular biology in sepsis: pathophysiological and therapeutic significance of vascular dysfunction J Smooth Muscle Res 2007 43 117 137 10.1540/jsmr.43.117 17928746

[B39] Zhou M Simms HH Wang P Adrenomedullin and adrenomedullin binding protein-1 attenuate vascular endothelial cell apoptosis in sepsis Ann Surg 2004 240 321 330 10.1097/01.sla.0000133253.45591.5b 15273558 PMC1356410

[B40] Rittirsch D Hoesel LM Ward PA The disconnect between animal models of sepsis and human sepsis J Leukoc Biol 2007 81 137 143 10.1189/jlb.0806542 17020929

[B41] Ertel W Morrison MH Wang P Ba ZF Ayala A Chaudry IH The complex pattern of cytokines in sepsis: association between prostaglandins, cachectin and interleukins Ann Surg 1991 214 141 148 10.1097/00000658-199108000-00008 1867521 PMC1358513

[B42] Wang P Ba ZF Chaudry IH Endothelium-dependent relaxation is depressed at the macro- and microcirculatory levels during sepsis Am J Physiol 1995 269 R988 R994 7503327 10.1152/ajpregu.1995.269.5.R988

[B43] Yang S Chung CS Ayala A Chaudry IH Wang P Differential alterations in cardiovascular responses during the progression of polymicrobial sepsis in the mouse Shock 2002 17 55 60 10.1097/00024382-200201000-00010 11795670

[B44] Grobmyer SR Armstrong RC Nicholson SC Gabay C Arend WP Potter SH Melchior M Fritz LC Nathan CF Peptidomimetic fluoromethylketone rescues mice from lethal endotoxic shock Mol Med 1999 5 585 594 10551900 PMC2230465

[B45] Sarkar A Hall MW Exline M Hart J Knatz N Gatson NT Wewers MD Caspase-1 regulates Escherichia coli sepsis and splenic B cell apoptosis independently of interleukin-1beta and interleukin-18 Am J Respir Crit Care Med 2006 174 1003 1010 10.1164/rccm.200604-546OC 16908867 PMC2648100

[B46] Conus S Perozzo R Reinheckel T Peters C Scapozza L Yousefi S Simon HU Caspase-8 is activated by cathepsin D initiating neutrophil apoptosis during the resolution of inflammation J Exp Med 2008 205 685 698 10.1084/jem.20072152 18299403 PMC2275389

[B47] Kennedy NJ Kataoka T Tschopp J Budd RC Caspase activation is required for T cell proliferation J Exp Med 1999 190 1891 1896 10.1084/jem.190.12.1891 10601363 PMC2195711

[B48] Greten FR Arkan MC Bollrath J Hsu LC Goode J Miething C Göktuna SI Neuenhahn M Fierer J Paxian S Van Rooijen N Xu Y O'Cain T Jaffee BB Busch DH Duyster J Schmid RM Eckmann L Karin M NF-kappaB is a negative regulator of IL-1beta secretion as revealed by genetic and pharmacological inhibition of IKKbeta Cell 2007 130 918 931 10.1016/j.cell.2007.07.009 17803913 PMC2134986

[B49] Yamada M Ichikawa T Ii M Sunamoto M Itoh K Tamura N Kitazaki T Discovery of novel and potent small-molecule inhibitors of NO and cytokine production as antisepsis agents: synthesis and biological activity of alkyl 6-(N-substituted sulfamoyl)cyclohex-1-ene-1-carboxylate J Med Chem 2005 48 7457 7467 10.1021/jm050623t 16279805

